# Separating the effects of air and soil temperature on silver birch. Part II. The relation of physiology and leaf anatomy to growth dynamics

**DOI:** 10.1093/treephys/tpac093

**Published:** 2022-08-08

**Authors:** Jouni Kilpeläinen, Timo Domisch, Tarja Lehto, Minna Kivimäenpää, Françoise Martz, Sirpa Piirainen, Tapani Repo

**Affiliations:** Natural Resources Institute Finland (Luke), Yliopistokatu 6 B, Joensuu 80100, Finland; Natural Resources Institute Finland (Luke), Yliopistokatu 6 B, Joensuu 80100, Finland; School of Forest Sciences, University of Eastern Finland, Yliopistokatu 7, 80100 Joensuu, Finland; Natural Resources Institute Finland (Luke), Latokartanonkaari 9, 00790 Helsinki, Finland; Department of Environmental and Biological Sciences, University of Eastern Finland, Yliopistonranta 1 E, 70210 Kuopio, Finland; Natural Resources Institute Finland (Luke), Juntintie 154, 77600 Suonenjoki, Finland; Natural Resources Institute Finland (Luke), Ounasjoentie 6, 96200 Rovaniemi, Finland; Natural Resources Institute Finland (Luke), Yliopistokatu 6 B, Joensuu 80100, Finland; Natural Resources Institute Finland (Luke), Yliopistokatu 6 B, Joensuu 80100, Finland

**Keywords:** Betula pendula, boreal trees, leaf morphology, leaf water potential, photosynthesis, plant acclimation, soil warming, tree ecophysiology

## Abstract

The aboveground parts of boreal forest trees grow earlier in the growing season, the roots mostly later. The idea was to examine whether root growth followed soil temperature, or whether shoot growth also demanded most resources in the early growing season (soil temperature vs internal sink strengths for resources). The linkage between air and soil temperature was broken by switching the soil temperature. We aimed here to identify the direct effects of different soil temperature patterns on physiology, leaf anatomy and their interactions, and how they relate to the control of the growth dynamics of silver birch (*Betula pendula* Roth).

Sixteen 2-year-old seedlings were grown in a controlled environment for two 14-week simulated growing seasons (GS1, GS2). An 8-week dormancy period interposed the GSs. In GS2, soil temperature treatments were applied: constant 10 °C (Cool), constant 18 °C (Warm), early growing season at 10 °C switched to 18 °C later (Early Cool Late Warm) and 18 °C followed by 10 °C (Early Warm Late Cool) were applied during GS2.

The switch from cool to warm enhanced the water status, net photosynthesis, chlorophyll content index, effective yield of photosystem II (Δ*F*/*F*_*m*′_) and leaf expansion of the seedlings. Warm treatment increased the stomatal number per leaf. In contrast, soil cooling increased glandular trichomes. This investment in increasing the chemical defense potential may be associated with the decreased growth in cool soil. Non-structural carbohydrates were accumulated in leaves at a low soil temperature showing that growth was more hindered than net photosynthesis. Leaf anatomy differed between the first and second leaf flush of silver birch, which may promote tree fitness in the prevailing growing conditions. The interaction of birch structure and function changes with soil temperature, which can further reflect to ecosystem functioning.

## Introduction

In boreal forests, it is common for tree root production to continue abundantly until the late growing season (Abramoff and Finzi 2015). This may be due to the soil warming in late summer (external driver of root growth) or the growth rhythms of different parts of trees, aboveground parts growing earlier and root growth mostly afterwards (internal driver). However, it is not possible to assess whether external or internal drivers are more important, because both vary in the same direction in field conditions. The idea of our experiment was to break the linkages between air and soil temperatures by switching the soil temperature between cool and warm in the middle of the growing season, without changing air temperature, the photoperiod or other factors, and then to monitor how tree growth and phenology were affected (Kilpeläinen et al., in press). Previously, we reported that the seedlings grew the largest in the constantly warm soil, but the treatments did not affect proportional growth allocation between shoots and roots (Kilpeläinen et al., in press). Soil cooling slowed down and soil warming accelerated shoot elongation. Short root mortality increased strongly with increasing soil temperature, but the timing of abundant root growth was not strongly driven by the applied soil temperature patterns. We concluded that soil temperature treatments did not solely drive the growth and phenological events of roots and shoots, but the tree-intrinsic drivers also played a significant role (Kilpeläinen et al., in press), and now we continue to explore the mechanisms.

The sink strengths of different tree parts for carbohydrates can vary during the annual cycle and can be greatly affected by growth-limiting factors such as soil temperature. According to previous studies, a low soil temperature (≤10 °C) prevailing throughout the growing season decreased chlorophyll content, net assimilation rate and stomatal conductance in silver birch (*Betula pendula* Roth) saplings (Aphalo et al. 2006). In aspen (*Populus tremuloides* Michx.) seedlings, a low soil temperature reduced root water flow, stomatal conductance, net assimilation and shoot water potential (Wan et al. 1999). Stomatal conductance, photosynthetic rate and the photochemical efficiency of foliage were suppressed in Norway spruce (*Picea abies* L. Karst.) at low soil temperatures (≤9 °C) (Lahti et al. 2002, Repo et al. 2004). If a low soil temperature suppresses net photosynthesis less than the sink strength of roots, non-structural carbohydrate (NSC) content can increase in leaves/needles, as was observed in Norway spruce (Repo et al. 2004). A relatively higher allocation to absorptive roots than to shoots in colder than warmer climates is generally observed (Ostonen et al. 2011, Zadworny et al. 2016). However, if other factors are not limiting, a low soil temperature decreases root growth, which leads to smaller root systems with smaller root surface areas and to a reduced potential for water and nutrient uptake, leading to decreased aboveground growth (Pregitzer et al. 2000, Yuan and Chen 2010).

Besides physiology and growth, soil temperature can affect the leaf anatomy of trees. Earlier studies have mostly dealt with the effects of air temperature or the combined effects of both air and soil temperature. In the previous studies with air warming using infrared heaters, apparently with concomitant soil warming too, the temperature increase was connected with lower glandular trichome densities of silver birch leaves (Thitz et al. 2017, Hartikainen et al. 2020), and with thinner and larger leaves in silver birch (Hartikainen et al. 2020) and European aspen (*Populus tremula* L.) (Hartikainen et al. 2009). Tree physiology, anatomy and growth are inherently connected. Adverse soil temperature can stress trees and therefore increase secondary metabolism through more numerous glandular trichomes (Oksanen 2018). A thicker palisade layer can be related to increased photosynthesis potential (Hartikainen et al. 2009). Thick leaves may diminish evapotranspiration (Larcher 2003) that would compensate the reduced water uptake in cold soil (Pallardy 2008). Cold tolerance can also increase with leaf thickness (Niinemets 2016). Marek et al. (2022) found that the stomatal density of Scots pine increased with mean annual temperature in a geographical gradient in Europe. The number of stomata is also probably affected by the soil temperature, and this can be reflected in gas exchange (Li et al. 2017). The trichome number is determined in the early stage of birch leaf development (Valkama et al. 2004). Silver birch has two leaf flushes, i.e., short-shoot leaves emerge from overwintered buds in the spring, and long-shoot leaves from the new buds of the current growing season. The anatomy of the leaf types can differ, because the previous year’s conditions affect short-shoot leaves more, and because climatic conditions in early and later seasons may differ and affect anatomy.

Silver birch is an economically and ecologically important widespread boreal tree species. As a species with high plasticity, it may benefit from climate warming although simultaneously, e.g., herbivory damage may increase, and early-stage survival may decrease at low fertility sites (Silfver et al. 2020, Oksanen 2021, Possen et al. 2021). Although the observed and anticipated air temperature rise will lead to warmer soils during growing season, winters will still be cold and a shorter time with insulating snowpack can lead to more frequent soil freeze–thaw cycles and lower soil temperature in future winters and springs (Halim and Thomas 2018), which will affect the functioning silver birch and boreal forest ecosystems.

We aimed here to test with a controlled environment experiment how the physiology (water and carbon relations, gas exchange) and leaf anatomy of silver birch (*B. pendula* Roth) are affected by different soil temperature patterns during the growing season, and how they are linked to the control of shoot growth and phenology. Two of our experimental treatments had constant soil temperatures (Cool, 10 °C and Warm, 18 °C) that were selected to be at the lower and higher end of the variability observed in the field during the growing season (Kubin and Kemppainen 1991). The lower soil temperature was suboptimal, and the higher temperature closer to optimal for root growth (Solfjeld and Johnsen 2006). In the third and fourth treatments, the soil temperature was switched from low to high and high to low, respectively, in the middle of the growing season (Early Cool Late Warm, ECLW and Early Warm Late Cool, EWLC). Among the treatments, ECLW most resembles the natural conditions. We hypothesized that (i) tree physiological traits in terms of leaf water potential, stem sap flow, leaf gas exchange, chlorophyll fluorescence and chlorophyll content are affected more negatively by soil cooling than warming; (ii) if a low soil temperature suppresses the root and shoot growth of silver birch more than photosynthesis, NSCs are accumulated; and (iii) leaf anatomy is affected by the changes in soil temperature alone (no change in air temperature) during the growing season so that structures associated with enhanced secondary and primary metabolism are connected with cool and warm soil, respectively. Our set-up also enabled the examination of responses in short-shoot and long-shoot leaves, and a corollary is therefore that, depending on growing conditions, silver birch may adjust the structure and functioning of the leaf types independently.

## Materials and methods

### Plant material and growing conditions

The growing conditions of silver birch (*B. pendula* Roth) seedlings are described in detail by Kilpeläinen et al. (in press). Briefly, 2-year-old seedlings were grown for two simulated growing seasons (GS1 and GS2) in four dasotrons (RTR48 Conviron controlled environment rooms) in the Joensuu Root Laboratory (Finér et al. 2001). Each dasotron had four containers and each container had one seedling. GS1 included an 11-week long-day phase (LD), and GS2 a 12-week LD. Both GSs included 3-week short-day phases (SD) after the LD. A 10-week dormancy period (D1) interposed the GSs. In GS1, the seedlings were acclimated to the dasotron conditions. Four soil temperature treatments were applied during GS2: constant 10 °C (Cool), constant 18 °C (Warm), early growing season at 10 °C switched to 18 °C later (ECLW) and 18 °C followed by 10 °C (EWLC). Soil temperature was changed gradually in ECLW and EWLC over 5 days in the middle of GS2 (46–50 days since the start of GS2); the middle day of GS2 is shown in figures. Hereafter, days refer to days since the start of a growing season, and the letters E and L with day numbers refer to early and late GS2, respectively. Other conditions than soil temperature were similar in all the treatments.

### Leaf area monitoring

The short-shoot leaves emerging from buds numbers 3, 5 and 7 counted from treetop downwards were photographed on a coordinate paper as a background, and the leaf area was assessed with the photo editing program ImageJ 1.51n (National Institutes of Health, Bethesda, MD, USA). The monitoring was continued until leaf growth ceased. Emerging long shoots at the upper third, middle and lower third of tree canopies (three leaves/tree) were monitored for leaf area as for short-shoot leaves. In addition, the area of leaves four and five from the top of the main shoot, and the area of leaves four and five from the top of the topmost branch was determined at the end of the SD period of GS2 (day L101). Leaf areas were averaged by tree.

### Chlorophyll fluorescence and chlorophyll content index

A fluorometer (Waltz PAM-2500, Heinz Walz Gmbh, Effeltrich, Germany) was used to measure chlorophyll fluorescence at five sampling times during GS2 from two short-shoot leaves in the upper third of the canopy. In addition, after emerging, two long-shoot leaves were measured similarly four times. Dark-acclimated (30 min) leaves were measured to assess the maximum photochemical quantum yield of photosystem II (*F*_v_/*F*_m_) (Baker 2008). Subsequently, light-acclimated leaves were measured after 3 min in 300 μmol m^−2^ s^−1^ for information on the quantum yield of photochemical quenching, i.e., the efficiency of light reactions in PSII (Δ*F*/*F*_m′_).

The chlorophyll content index (CCI) was measured weekly during GS2 from three short- and long-shoot leaves with a portable meter (CCM-300, Opti-Sciences, Hudson, NH, USA). The values of *F*_v_/*F*_m_, Δ*F*/*F*_m′_ and CCI were averaged by leaf type for further testing.

### Leaf water potential

Leaf water potential (ψ) was measured from two leaves from the upper third of the canopy of each seedling five times during GS2 with a pressure chamber (Model 1000, PMS Instruments, Albany, OR, USA). Short-shoot leaves were sampled at E29 and E43 days, and long-shoot leaves at L57, L78 and L92 days since the start of GS2. Each leaf was measured immediately after cutting, and the measurements were carried out within 2 h around midday. The averaged ψ by tree was used for statistical analyses.

### Stem sap flow

Stem sap flow was continuously monitored (data saved at 15-min intervals) with a sap flow gauge system (Dynamax Flow32, Dynamax Inc., Houston, TX, USA). The SGB9-WS gauges were installed 5 cm above the root collars. The stem surfaces were cleaned and smoothed with natural oil, and the inside surfaces of the gauges were greased thinly (Electrical insulating compound 4, Dow Corning Co., Midland, MI, USA). The power input was 0.13 W. In sap flow calculation, the thermal conductance was calculated as the mean value within 2 h before the lights were switched on in the morning (Dynamax 2017). The threshold for temperature difference between the upper and lower thermocouple in the gauge was set to 0.3 °C, and below this, the sap flow rate was assumed to be zero. The sap flow rate was recorded at 15-min intervals, and the daily flow was summed for further analysis.

### Gas exchange measurements

Net photosynthesis, stomatal conductance and transpiration were determined five times during GS2, first in the LD conditions and the latest measurements in SD conditions. A portable photosynthesis system was used (LI-6400XT, LI-COR Inc., Lincoln, NE, USA) at light saturation (900 μmol m^−2^ s^−1^). Light response curves (measured during GS1 before the first measurements, data not shown) were used to confirm that photosynthesis was light saturated at the selected measuring irradiance. Measurements were conducted during the morning hours (09–12) from one leaf inside the canopy and emerging from the stem (‘old’), and from one leaf emerging later from a branch (‘new’). Measurements were taken only for leaves that were sufficiently large to cover the measurement area of the cuvette (6 cm^2^). The applied CO_2_ concentration was 400 ppm and block temperature was 20 °C. The last measurement during GS2 SD on three old leaves in the Warm treatment was conducted on different leaves than in the previous measurements, because they had fallen. The light-saturated net photosynthesis rate (*A*_max_), stomatal conductance (*g*_s_), transpiration (*E*), intercellular CO_2_ concentration (*C*_i_) and instantaneous water use efficiency (WUE) as *A*_max_/*E* ratio were computed. Respiration (*R*) rates were measured after switching PAR to zero.

### NSCs

Two to three short-shoot leaves were collected on days E31and E44, and two to three of both short-shoot and long-shoot leaves on days L58, L78 and L91 for NSCs, whereas the stem parts and roots were separated at final harvest (day L134) only. All plant parts were frozen at −80 °C immediately after separation for further analyses later as described by Domisch et al. (2018). The samples were freeze-dried (Christ Alpha 1–4 LD, Martin Christ Gefriertrocknungsanlagen GmbH, Osterode, Germany), ball-milled (Fritsch Pulverisette 23, Fritsch GmbH, Idar-Oberstein, Germany) and stored at −20 °C until analysing. Soluble sugars were extracted from 15–20 mg of dry milled tissue, three times with 0.5 ml water at 80 °C, and analysed by high-performance liquid chromatography (NexeraX2, Shimadzu Co, Kyoto, Japan) using a ligand exchange column (Hi-Plex Ca, 300 × 7.7 mm, Agilent, Santa Clara, CA, USA). Elution was done with MilliQ water at 80 °C (0.6 ml min^−1^) and detection with an evaporative light scattering detector (Sedex 90LT, Sedere, Olivet, France). Glucose, sucrose and α-pinitol (Sigma-Aldrich Co., St Louis, MO, USA), fructose, raffinose and meso-erythritol (ThermoFisher Scientific, Waltham, MA, USA) were used for calibration. Starch in the remaining pellet was hydrolyzed enzymatically, and the glucose produced was quantified using the glucose oxidase/peroxidase method (K-GLUC, Megazyme), adapted to the microplate. In the results, glucose, sucrose and fructose summed up soluble sugars, and NSC additionally included starch.

### Leaf anatomy

Leaves were sampled twice during GS2. The first sampling time was on day E35 (2 weeks before the temperature switch in ECLW and EWLC), and the second on day L77 (4 weeks after the temperature switch). Four short-shoot leaves (grown from old buds) were collected in the lower part of the upper canopy third of each seedling at the first sampling time. At the second sampling time, four long-shoot leaves (grown from current buds after the soil temperature switch) of the same age as in the first sampling were collected in the same way as in the first sampling. Two leaves of each seedling were used to study the upper and lower leaf surfaces, and the leaf replicas were prepared immediately after the sampling. Two other leaves were sampled and refrigerated in fixative (2% glutaraldehyde in cacodylate buffer, 0.05 M) for further preparation and analyses of the inner anatomy.

To calculate the density of the stomata, and glandular and non-glandular trichomes, and to determine epidermis cell areas, the leaf surface structure from the leaf edge to the central vein between two side veins in the middle of each leaf was copied with clear nail polish to transparent tape from both sides of the leaf (Wang et al. 2016). The leaf replicas were placed on microscope slides, and the area from the leaf edge to the central vein was divided visually into three parts. Photographs were taken from the three parts with a digital camera (Leica DFC295, Leica microsystems, Wetzlar, Germany) under a light microscope (Leica DM2500) with ×20 objective magnification to count stomata and to determine average epidermis cell area (these were counted only at the lower leaf surface), and with ×10 objective magnification to count glandular and non-glandular trichomes. Glandular trichomes were characterized by lighter outer cells and darker base, as shown by Valkama et al. (2004), and non-glandular trichomes by hairs pointing outwards from the marguerite-like base structure, as described by Wang et al. (2016). For each leaf, the densities of stomata and trichomes per unit image area were averaged from the three photographed leaf parts, and the total numbers of stomata and trichomes per leaf were calculated based on total leaf area. In addition, the indices of trichomes were calculated as the number of trichomes per unit area/(number of epidermal cells per unit area + trichomes per unit area) × 100 (Dilcher 1974). These indices were calculated only for the lower leaf side, because the epidermal cells were investigated only on this side. Average values by tree by leaf side were applied in the further testing of leaf anatomy traits. Leaves were photographed on a millimeter paper, and the total leaf area was determined. The images were analysed with tools of ImageJ 1.47v (Abràmoff et al. 2004).

A leaf sample with an area of ca. 1.5 × 1.5 mm^2^ was taken to study the inner anatomy near the central vein and between two side veins in the middle of each sample leaf. The samples were prepared following Sutinen et al. (1990, 2015), and cross-sections of them were cut with a microtome (Leica EM UC7) for microscopic imaging (the same equipment as above) with ×20 objective magnification. The thickness of leaf, upper epidermis, lower epidermis, and the palisade and spongy layers were determined from three points in the images with ImageJ 1.47v. The palisade–spongy ratio was calculated. Average values by tree were applied for statistical analyses.

### Statistical analyses

Two soil temperatures prevailed before the soil temperature switch, but the tests were run as if all four treatments were already present. In the linear mixed models of leaf area and leaf water potential, seedling was a subject and sampling time a repeated variable (covariance type AR1), and soil temperature treatment, sampling time and their interaction were fixed factors. Short-shoot and long-shoot leaves were tested separately. In the linear mixed models of gas exchange results, seedling was a subject and sampling time a repeated variable (covariance type AR1), and soil temperature treatment, sampling time, leaf age and their interaction were fixed factors, whereas seedling was a random factor. In the linear mixed models of chlorophyll fluorescence and NSC content of short-shoot and long-shoot leaves, seedling was a subject and sampling time a repeated variable (covariance type AR1), and soil temperature treatment, sampling time and their interaction were fixed factors. Leaf anatomy variables as well as NSC in tree parts and long-shoot leaf area at the end of the experiment were tested with one-way analysis of variance for treatment effects. The trichome and stoma variables were tested with a paired samples *t*-test for leaf type differences. Stem sap flow was tested separately before and after the soil temperature switch with a similar linear mixed model, except time was a covariate to test trends in the daily values by treatment. Because CCI, which was also densely (weekly) measured, showed significantly different trends before and after the soil temperature switch, it was tested with a similar linear mixed model, as in the sap flow before and after the soil temperature switch. The post hoc tests were Bonferroni-adjusted pairwise comparisons. These analyses were run with IBM SPSS Statistics 27.0.0.0 software (IBM, Armonk, NY, USA). In the results, the significance probabilities are denoted with subscripts T for temperature treatment, t for sampling time and adj for the Bonferroni comparison.

## Results

### Leaf area

**Figure 1.**
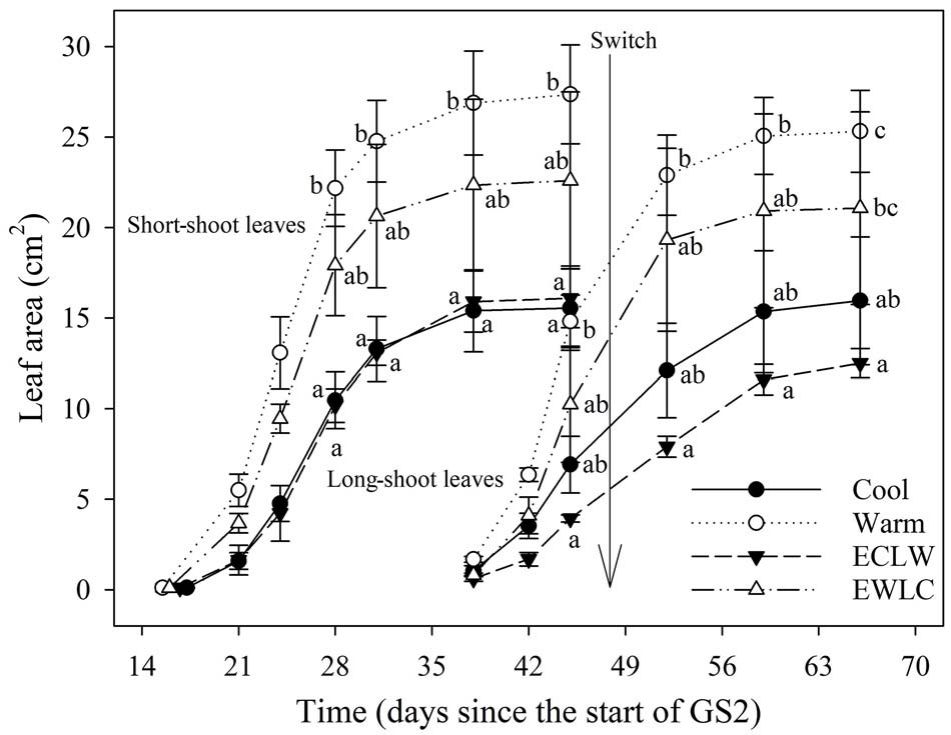
Mean leaf area (±SE) of silver birch seedlings (*n* = 4) during the growing season GS2 with soil temperature treatments (Cool = constant 10 °C, Warm = constant 18 °C, ECLW = Early Cool Late Warm, EWLC = Early Warm Late Cool). The arrow indicates the time of the temperature switch in ECLW and EWLC. The curves on the left represent short-shoot leaves, and on the right, long-shoot leaves. The different letters indicate significant differences between treatments within a measuring time (*P*_adj_ ≤ 0.05).

In GS2, the areas of the first leaves from both the first (short-shoot leaves) and the second flush (long-shoot leaves) grew largest in Warm and EWLC, and smallest in ECLW and Cool. The short-shoot leaf area was higher in Warm than in Cool and ECLW after day E28 (E and L refer to early and late growing season before and after the soil temperature switch, respectively) (Figure 1). Long-shoot leaf area was larger in Warm than ECLW after day E45, and larger in Warm than Cool, and larger in EWLC than ECLW, on day L52 (Figure 1).

As the monitored long-shoot leaves started to grow before the soil temperature change, the change did not significantly affect the long-shoot leaf area, and the growth pattern remained nearly similar to that in short-shoot leaves (Figure 1). However, the effect of soil temperature change was more visible in the long-shoot leaves that developed after the change and were measured at the end of GS2. These leaf areas were 26.7 ± 6.4, 25.0 ± 2.9, 41.4 ± 3.0 and 20.0 ± 2.5 cm^2^ in Cool, Warm, ECLW and EWLC, respectively, and the leaves in ECLW were significantly larger than in EWLC (*P*_T_ = 0.015, *P*_adj_ = 0.015).

### Leaf water potential and stem sap flow

**Figure 2.**
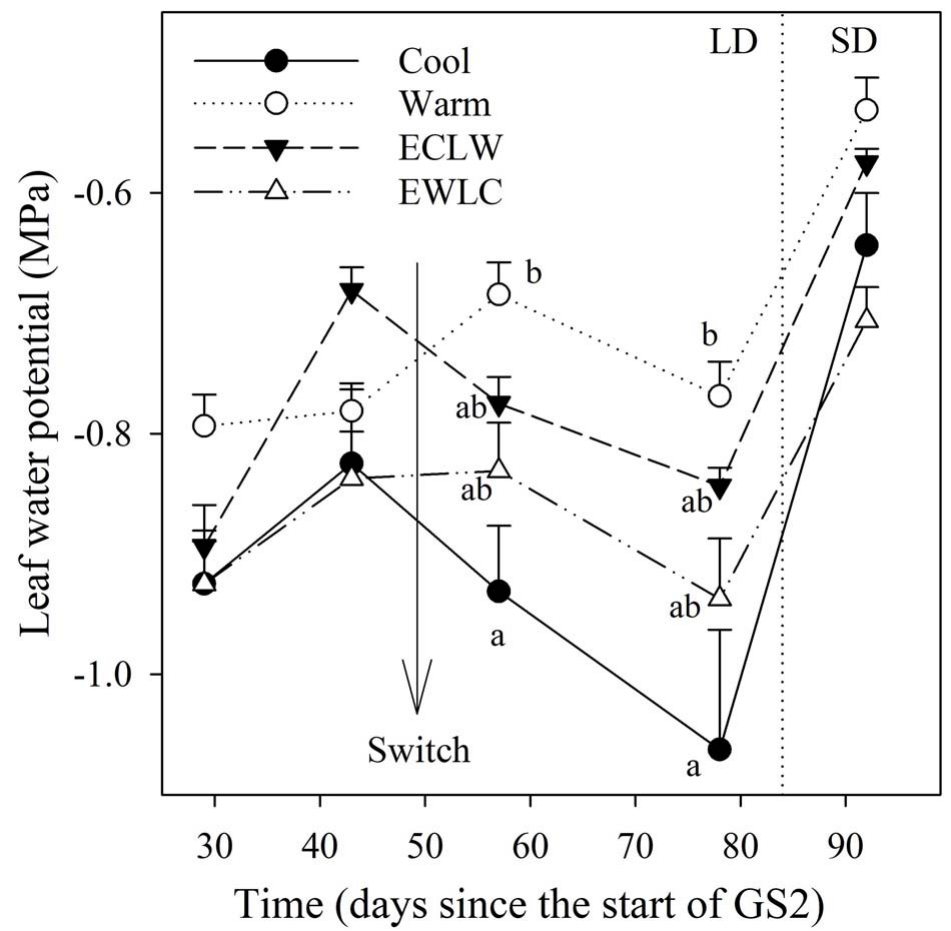
Leaf water potential of silver birches grown in different soil temperature treatments (Cool = constant 10 °C, Warm = constant 18 °C, ECLW = Early Cool Late Warm, EWLC = Early Warm Late Cool). The arrow indicates the time of the temperature switch in ECLW and EWLC. Short-shoot leaves were measured at times 1–2, and long-shoot leaves at times 3–5. Bars show standard errors of means (*n* = 4). The different letters indicate significant differences between treatments within a measuring time (*P*_adj_ ≤ 0.05).

Significant treatment effects on leaf water potential (ψ) were only observed during the second half of GS2, after the temperature switch: constantly cool soil led to lower ψ than constantly warm soil, with intermediate values measured in treatments where the temperature was switched (Figure 2, Table 1). The ψ of long-shoot leaves was lower in Cool than in Warm on days L57 and L78. No effect was observed thereafter in SD conditions (last measurement on day L92).

**Table 1:** *P*-values of statistical tests (zero omitted before decimal separator) of the leaf area monitoring, leaf water potential (ψ), stem sap flow, chlorophyll fluorescence (*F*_v_/*F*_m_, Δ*F*/*F*_m′_) and chlorophyll content index (CCI).

Trait	Treatment	Time	Treatment × Time
SSL area	**.015**	**<.001**	**.012**
LSL area	**.049**	**<.001**	**<.001**
SSL ψ	.244	**.028**	.428
LSL ψ	**.024**	**<.001**	.592
Sap flow, early	.770	**<.001**	.097
Sap flow, late	**<.001**	.789	**<.001**
SSL *F*_v_/*F*_m_	.992	**<.001**	**<.001**
LSL *F*_v_/*F*_m_	**.035**	**<.001**	.270
SSL Δ*F*/*F*_m′_	**.004**	**<.001**	**<.001**
LSL Δ*F*/*F*_m′_	.307	**<.001**	.466
SSL CCI, early	.843	**<.001**	.972
SSL CCI, late	**<.001**	**<.001**	**<.001**
LSL CCI, late	**.020**	**<.001**	**.002**

SSL, short-shoot leaf; LSL, long-shoot leaf. Early and late refer to measurements before and after the soil temperature switch, respectively. Values of *P* ≤ 0.05 are in bold. Post hoc test results are shown in respective figures.

**Figure 3.**
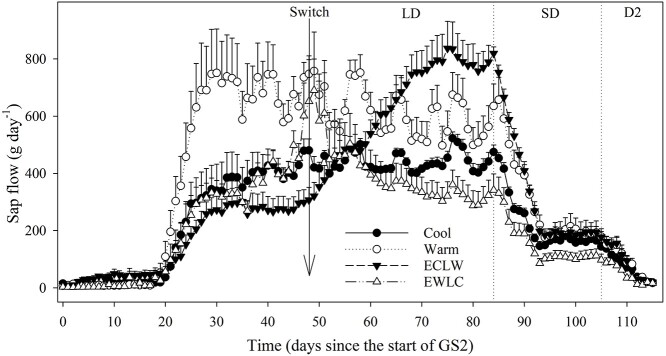
Stem sap flow (daily mean + SE, *n* = 4 except for Warm, *n* = 3 since day 92) of silver birch seedlings during growing season GS2, with different soil temperature treatments (Cool = constant 10 °C, Warm = constant 18 °C, ECLW = Early Cool Late Warm, EWLC = Early Warm Late Cool). The arrow indicates the time of the temperature switch in ECLW and EWLC. LD, long-day phase; SD, short-day phase; D2, dormancy period.

Stem sap flow started to increase rapidly around day E20 with leaf-out (Figure 3, cf. Figure 1). After this, until the soil temperature switch starting at E46, the sap flow was largest in Warm, where it increased more than in ECLW (estimate of interaction effect *P* = 0.012), whereas Cool and EWLC remained intermediate (Figure 3, Table 1). The effect of the soil temperature switch on sap flow was significant, as the flow increased with soil warming in ECLW and decreased with cooling soil in EWLC (ECLW differed from others and EWLC differed from Warm, estimates of interaction effects *P* ≤ 0.012) (Figure 3, Table 1). Concurrently, there was no clear change in Cool and Warm treatments before the start of the SD period (day L83), when the sap flow started to decrease rapidly in all treatments (Figure 3).

### Gas exchange, chlorophyll fluorescence and CCI

**Figure 4.**
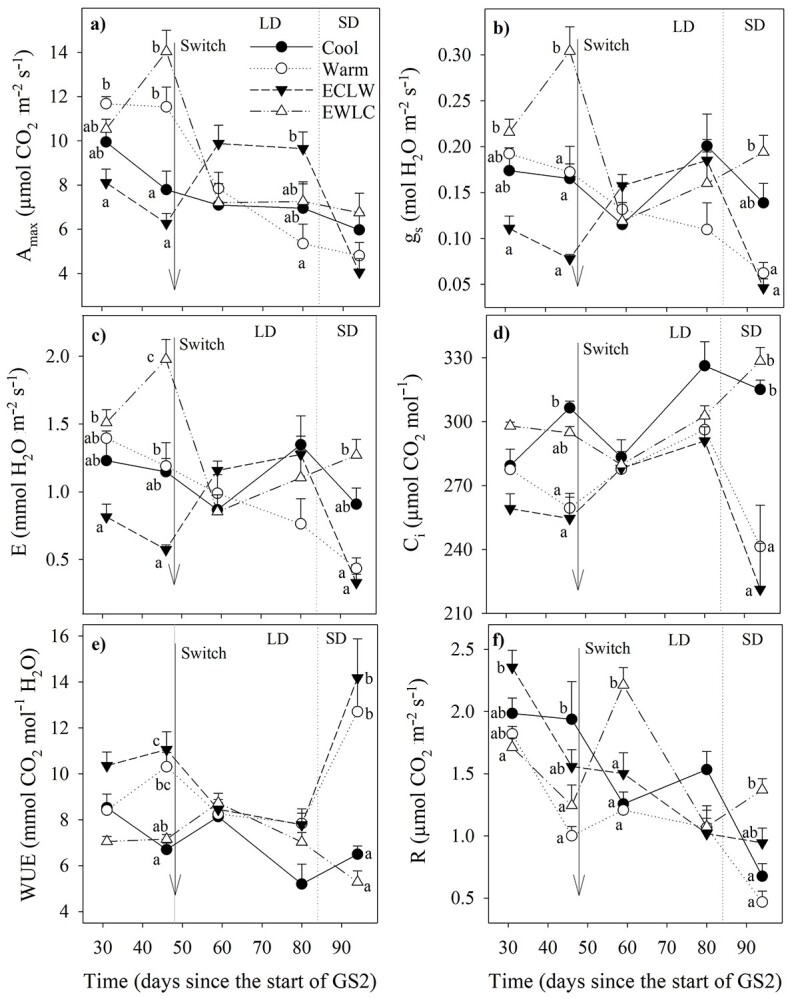
Light-saturated net assimilation rate (*A*_max_) (a), stomatal conductance (*g*_s_) (b), transpiration (*E*) (c), intercellular CO_2_ concentration (*C*_i_) (d), water use efficiency (WUE) (*e*) and respiration (*R*) (f) of silver birch in different soil temperature treatments (Cool = constant 10 °C, Warm = constant 18 °C, ECLW = Early Cool Late Warm, EWLC = Early Warm Late Cool). The arrow indicates the time of the temperature switch in ECLW and EWLC. Bars show standard errors of means (*n* = 4). LD, long-day phase; SD, short-day phase. The different letters indicate significant differences between treatments within a measuring time (*P*_adj_ ≤ 0.05).

Net photosynthesis (*A*_max_) changed with soil temperature. Before the soil temperature switch, *A*_max_ was higher in warm than in cool soil; after the switch, it increased in ECLW and decreased, respectively, in EWLC (Figure 4a, Table 2). At the same time, Cool showed no large difference, but for some reason, *A*_max_ in Warm showed a decreasing trend even before the start of SD that induced a decrease in all treatments.

The treatment effects were quite similar in stomatal conductance (*g*_s_) (Figure 4b) to transpiration (*E*) (Figure 4c). They were highest in EWLC and lowest in ECLW before the soil temperature switch, Cool and Warm being intermediate (Table 2). After the switch, they increased in ECLW and decreased in EWLC. ECLW showed the most rapid decline in *g*_s_ and *E*, as well as in intercellular CO_2_ concentration *C*_i_ (Figure 4d) in SD. The switch effect was visible in *C*_i_ in both ECLW and EWLC. In WUE (WUE = *A*_max_/*E*), the switch effect was the opposite, as changes in *E* were somewhat larger than in *A*_max_ (Figure 4e). Before the soil temperature switch, respiration (*R*) was higher in cool than warm soil, and *R* increased with the switch in EWLC (Figure 4f, Table 2).

New leaves had 31–41% higher *A*_max_, *g*_s_ and *E* than old leaves, and the difference was similar in all treatments. *C*_i_ was 4–21% lower, whereas WUE was 13–41% higher, in new leaves than in old leaves in ECLW after the second measuring time. *R* did not differ with leaf type. For clarity, gas exchange results were combined in Figure 4.

**Figure 5.**
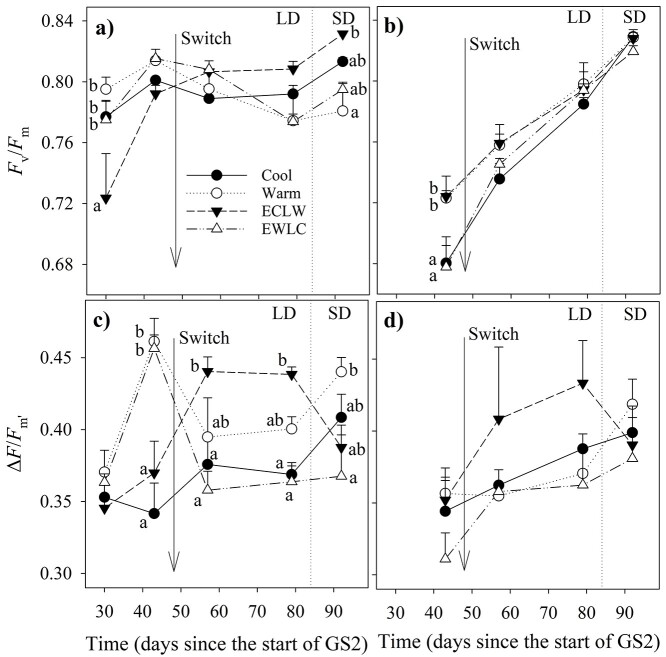
Photochemical efficiency (*F*_v_/*F*_m_) of (a) short-shoot and (b) long-shoot leaves, and effective yield of photosystem II (Δ*F*/*F*_m′_) of (c) short-shoot and (d) long-shoot leaves during the treatment growing season (GS2) (Cool = constant 10 °C, Warm = constant 18 °C, ECLW = Early Cool Late Warm, EWLC = Early Warm Late Cool). The arrow indicates the time of the temperature switch in ECLW and EWLC. Values are means + SE (*n* = 4). LD, long-day phase; SD, short-day phase. The different letters indicate significant differences between the treatments within a measurement session (*P*_adj_ ≤ 0.05).

Short-shoot leaf *F*_v_/*F*_m_ increased slightly in ECLW; it decreased in EWLC after the soil temperature switch, but Cool and Warm showed no significant difference (Figure 5a, Table 1). Long-shoot leaf *F*_v_/*F*_m_ increased towards the end of the growing season in all treatments, but the temperature switch had no effect (Figure 5b, Table 1). Short-shoot leaf Δ*F*/*F*_m′_ was higher in warm (Warm, EWLC) than cool (Cool, ECLW) soil before the soil temperature switch, but after the switch, it increased significantly in ECLW, whereas the opposite occurred in EWLC, and the initial order in Warm and Cool remained intermediate (Figure 5c, Table 1). Long-shoot leaf Δ*F*/*F*_m′_ showed no treatment differences (Figure 5d, Table 1).

**Figure 6.**
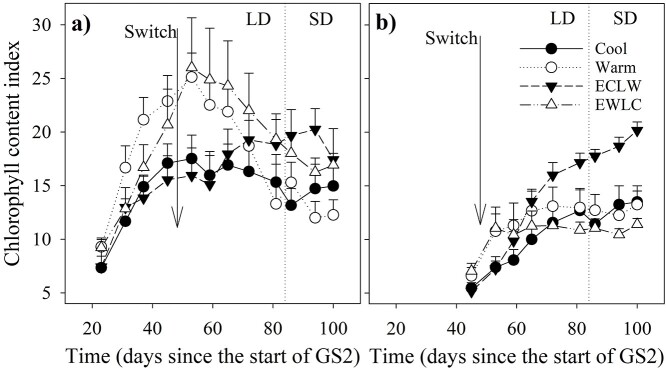
Mean (+SE, *n* = 4) chlorophyll content index of (a) short-shoot leaves and (b) long-shoot leaves in silver birch seedlings during the treatment growing season GS2 (Cool = constant 10 °C, Warm = constant 18 °C, ECLW = Early Cool Late Warm, EWLC = Early Warm Late Cool). The arrow indicates the time of the temperature switch in ECLW and EWLC. LD, long-day phase; SD, short-day phase.

CCI of short-shoot leaves showed a relation to soil temperature, as it increased most in Warm and EWLC and least in ECLW and Cool before the soil temperature switch, but the treatment differences were non-significant (Figure 6a, Table 1). CCI of short-shoot leaves increased after the switch in ECLW, whereas it decreased significantly in EWLC (estimates of interaction effects *P* ≤ 0.018) (Figure 6a, Table 1). However, Warm also showed a clear decline in CCI towards the end of GS2, and Cool also showed a slight decrease. The CCI of long-shoot leaves increased most towards the end of the growing season in the treatment ECLW (*P* < 0.001, *P* = 0.005 and *P* = 0.078 for estimates of EWLC × time, Warm × time and Cool × time compared with ECLW × time) (Figure 6b, Table 1).

### Leaf anatomy

**Figure 7.**
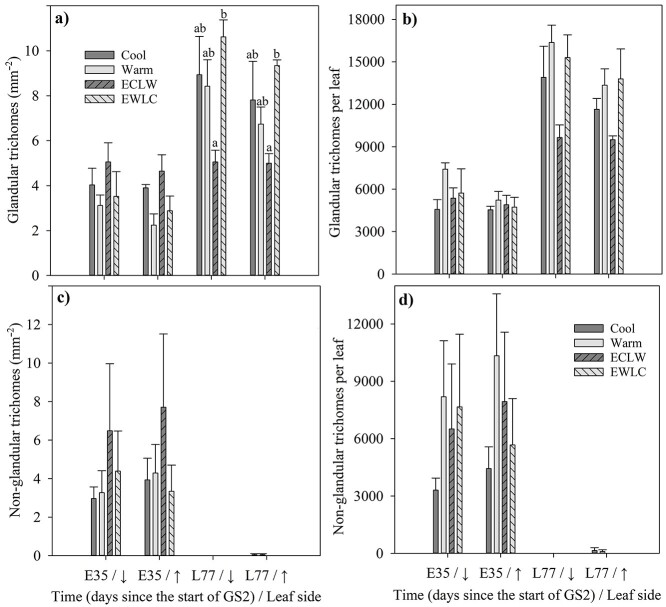
Glandular (a) and non-glandular trichome (b), density and number of glandular (c) and non-glandular trichomes (d) on lower (↓) and upper (↑) leaf surfaces of silver birches in soil temperature treatments. Short-shoot leaves were sampled on day E35 and long-shoot leaves on day L77 during treatment growing season GS2. The soil temperature was interchanged between cool (10 °C) and warm (18 °C) in treatments ECLW (early 10 °C, late 18 °C) and EWLC (early 18 °C, late 10 °C) between sampling times. The bars show mean + SE (*n* = 4). The different letters indicate significant differences between treatments (*P*_adj_ ≤ 0.05).

The glandular trichome density of short-shoot leaves tended to be higher in cool (Cool + ECLW) than warm (Warm + EWLC) soil before the soil temperature switch on day E35 (*P*_T_ = 0.412 and *P*_T_ = 0.043 for lower and upper leaf sides, respectively) (Figure 7a). The soil temperature switch was reflected in glandular trichomes, as their density on both lower and upper surfaces of long-shoot leaves was lower in ECLW than in EWLC after the switch on day L77 (*P*_T_ ≤ 0.030 and 0.049, *P*_adj_ = 0.028 and 0.050, respectively) (Figure 7a). Simultaneously, the glandular trichome density remained intermediate in Cool and Warm (Figure 7a). A similar pattern to that in glandular trichome density was also seen in the number of glandular trichomes per leaf in ECLW and EWLC after the switch (*P*_T_ = 0.047) (Figure 7b). However, the leaf-wise glandular trichome number was about the same in Warm as in ECLW, whereas it was intermediate in Cool on day L77 (pairwise comparisons were non- significant, *P*_adj_ ≥ 0.061) (Figure 7b). Glandular trichome numbers per leaf did not show treatment differences on day E35 (*P*_T_ ≥ 0.296). At the same time, non-glandular trichome densities per unit leaf area or total leaf area did not differ significantly between soil temperature treatments either (*P*_T_ ≥ 0.481) (Figure 7c and d). Only very few non-glandular trichomes were observed in long-shoot leaves on day L77 (Figure 7c and d). The non-glandular trichome index could be calculated only for short-shoot leaves sampled on day E35, and it did not differ between treatments (*P*_T_ = 0.788). In general, glandular trichomes were more numerous, whereas there were fewer non-glandular trichomes in long-shoot than short-shoot leaves (*P* < 0.001).

**Figure 8.**
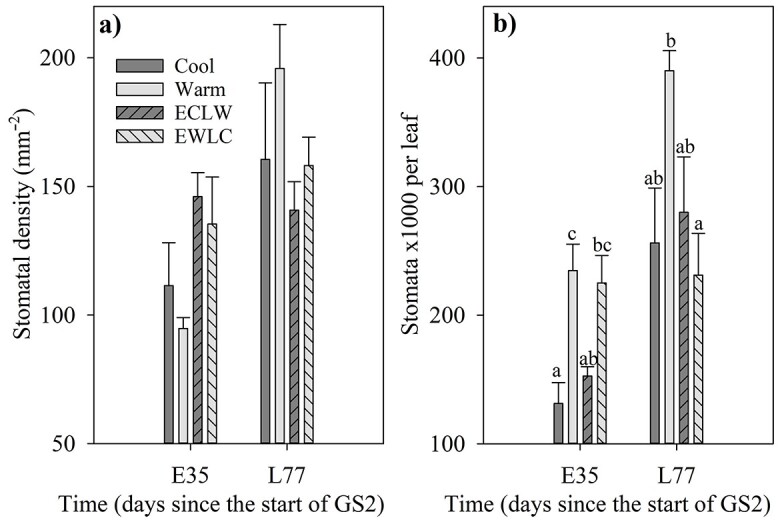
Stomatal density per unit leaf area (a) and number of stomata per leaf (b) at the lower surface of silver birch leaves. Short-shoot leaves were sampled on day E35, and long-shoot leaves on day L77. The soil temperature was interchanged between cool (10 °C) and warm (18 °C) in treatments ECLW (early 10 °C, late 18 °C) and EWLC (early 18 °C, late 10 °C) between sampling times. The bars show mean + SE (*n* = 4). The different letters indicate significant differences between treatments (*P*_adj_ ≤ 0.05).

Stomatal density per unit leaf area did not differ significantly between treatments in either of the sampling times (*P*_T_ ≥ 0.073) (Figure 8a). On day E35, the stomatal number per leaf was higher in currently warm (Warm, EWLC) than in currently cool (Cool, ECLW) treatments (*P*_T_ = 0.002, *P*_adj_ ≤ 0.034, for EWLC vs ECLW *P*_adj_ = 0.071), and on day L77, it was higher in Warm than in EWLC (*P*_T_ = 0.036, *P*_adj_ = 0.047) (Figure 8b). Stomatal density (*P* = 0.004) and number per leaf (*P* < 0.001) were on average higher in long-shoot than short-shoot leaves.

The mean area of short-shoot leaves collected on day E35 was highest in Warm (*P*_T_ < 0.001, *P*_adj_ ≤ 0.018), but the area of long-shoot leaves sampled 6 weeks later did not differ significantly between treatments (*P*_T_ = 0.507) (Table 3). On day L77, the average glandular trichome index was marginally higher in EWLC than in ECLW treatment (*P*_T_ = 0.070, *P*_adj_ = 0.087), but no differences were observed on day E35 (*P*_T_ = 0.387) (Table 3). On day L77, the lower epidermis was thinnest in Cool (*P*_T_ < 0.001, *P*_adj_ ≤ 0.023; on day E35, *P*_T_ = 0.197) (Table 3). On day L77, the spongy layer was thicker in ECLW than in the EWLC treatment (*P*_T_ = 0.048, *P*_adj_ = 0.045; on day E35, *P*_T_ = 0.368) (Table 3). Epidermis cell area (*P*_T_ ≥ 0.125), leaf thickness (*P*_T_ ≥ 0.343), upper epidermis thickness (*P*_T_ ≥ 0.604), palisade thickness (*P*_T_ ≥ 0.685) and palisade–spongy ratio (*P*_T_ ≥ 0.219) showed no treatment differences.

**Table 2:** *P*-values of statistical tests of the gas exchange results (zero omitted before decimal separator).

Trait	Treatment	Time	Treatment × Time	Leaf type	Treatment × Leaf type	Time× Leaf type	Treatment × Time ×Leaf type
*A*_max_	**.279**	**<.001**	**<.001**	**<.001**	.976	.376	.833
*g*_s_	**.044**	**<.001**	**<.001**	**<.001**	.299	.088	.924
*E*	**.042**	**<.001**	**<.001**	**<.001**	.328	.072	.927
*C*_i_	**.005**	**<.001**	**<.001**	**.020**	**.038**	**.045**	.856
WUE	**.004**	**<.001**	**<.001**	**.028**	**.043**	**.021**	.854
*R*	**.002**	**<.001**	**<.001**	.056	.244	.337	.559

Values of *P* ≤ 0.05 are in bold. Post hoc test results are shown in respective figures.

### NSCs

**Figure 9.**
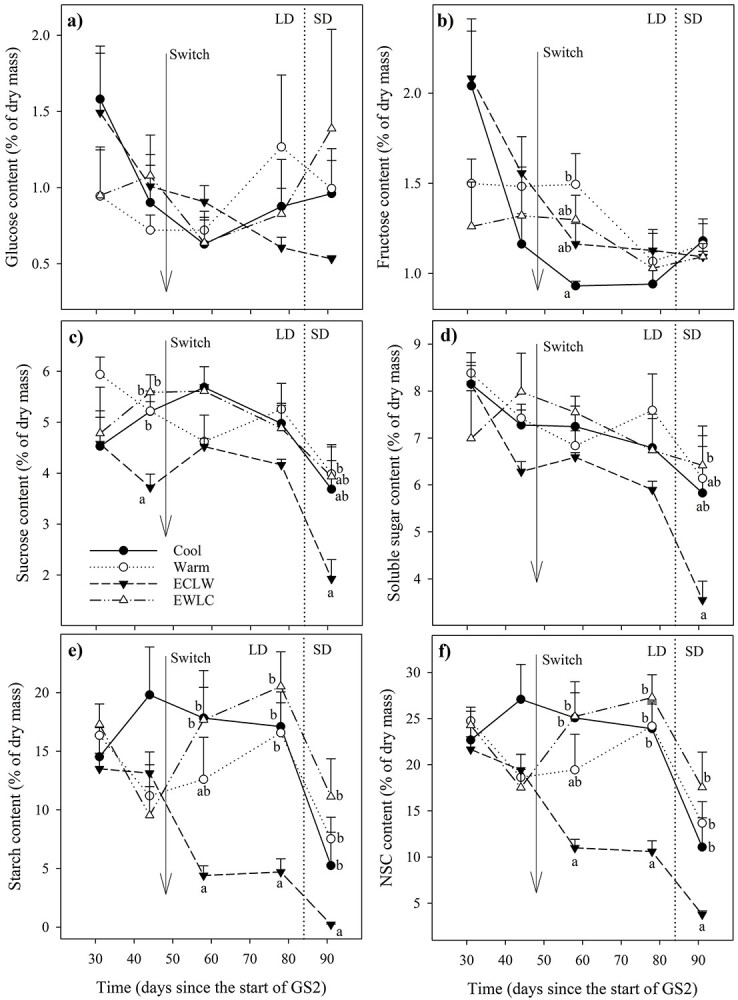
Carbohydrate content of short-shoot leaves of silver birch seedlings during the treatment growing season GS2 (Cool = constant 10 °C, Warm = constant 18 °C, ECLW = Early Cool Late Warm, EWLC = Early Warm Late Cool). The arrow indicates the time of the temperature switch in ECLW and EWLC. Values are means + SE (*n* = 4). LD, long-day phase; SD, short-day phase. The different letters indicate significant differences between the treatments within a measurement session (*P*_adj_ ≤ 0.05).

**Figure 10.**
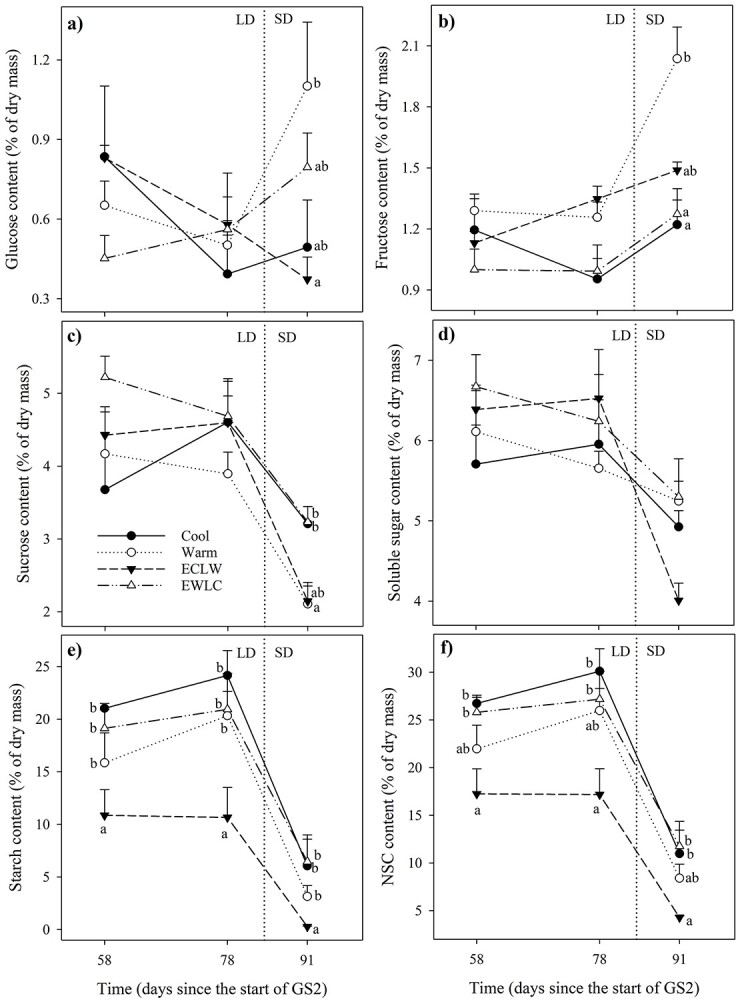
Carbohydrate content of long-shoot leaves of silver birch seedlings during treatment growing season GS2 (Cool = constant 10 °C, Warm = constant 18 °C, ECLW = Early Cool Late Warm, EWLC = Early Warm Late Cool). The arrow indicates the time of the temperature switch in ECLW and EWLC. Values are means + SE (*n* = 4). LD, long-day phase; SD, short-day phase. The different letters indicate significant differences between the treatments within a measurement session (*P*_adj_ ≤ 0.05).

**Figure 11.**
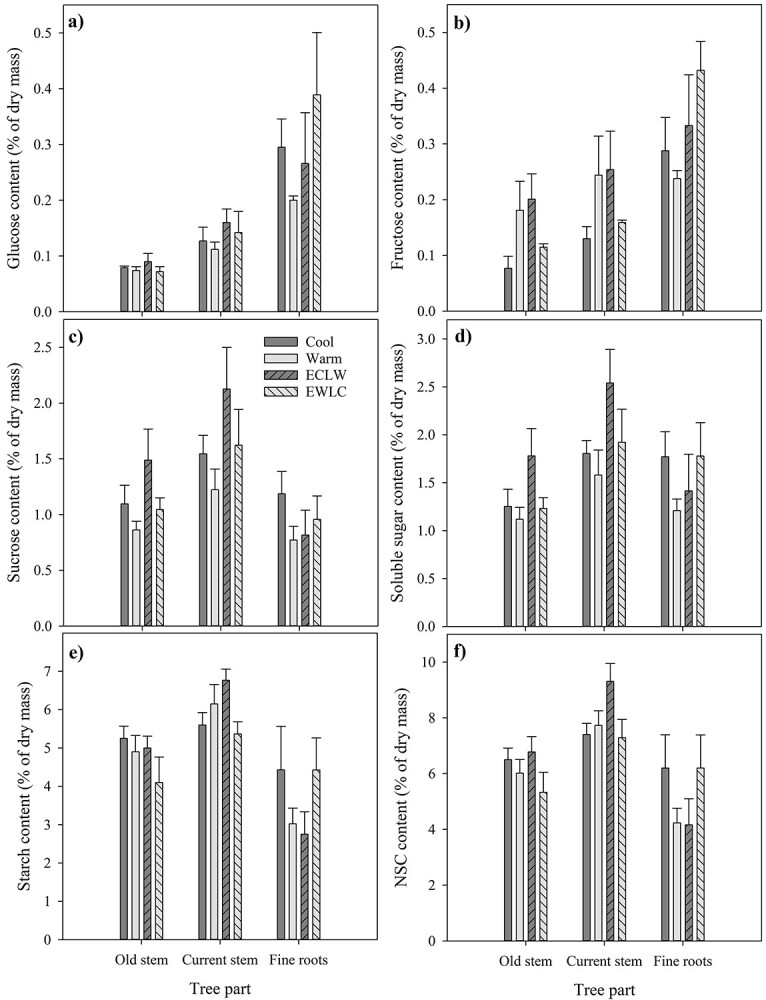
Carbohydrate content of the stems of the previous and current growing season (Old and Current, respectively) and of fine roots at final harvest of silver birch seedlings grown in different soil temperature treatments during GS2 (Cool = constant 10 °C, Warm = constant 18 °C, ECLW = Early Cool Late Warm, EWLC = Early Warm Late Cool).

The glucose content of short-shoot leaves did not differ significantly between treatments or times (Figure 9a, Table 4). The fructose content of short-shoot leaves was lower in Cool than in Warm on day L58 (Figure 9b). Sucrose was the major soluble sugar in all tissues (Figures 9–11) and so the soluble sugar content reflected its variation. The sucrose (Figure 9c) and total soluble sugar content (Figure 9d) was lower in ECLW than in EWLC on day L91. In ECLW, the content on day L91 was lower than in the earlier measurements. In ECLW, the sucrose (Figure 9c) and total soluble sugar content (Figure 9d) of short-shoot leaves was lowest on day L91 (*P*_adj_ ≤ 0.007). The starch (Figure 9e) and total NSC content (Figure 9f) in short-shoot leaves decreased most in ECLW and increased most in EWLC after the soil temperature switch (Table 4).

In long-shoot leaves, Warm and ECLW had low sucrose contents at the end of GS2 (L91, Figure 10c, Table 4). Those leaves had high glucose and fructose contents (Figure 10a and b). The starch (Figure 10e) and NSC content (Figure 10f) in long-shoot leaves was lower in ECLW than in the other treatments (Table 4), and lower in the last sampling than in the earlier ones. In both types of leaves, the total content of soluble sugars slowly decreased over the growing season, with a faster decline towards the end of GS2, and especially after the change to SD conditions (Figures 9d and 10d). In general, ECLW differed most prominently from the other treatments in most leaf carbohydrate content.

The largest treatment difference in the NSC content of the stems was found in the fructose content of the stem from the previous growing season, which was slightly higher in ECLW than in Cool (*P*_T_ = 0.053, *P*_adj_ = 0.072) (Figure 11b). The other contents of the previous or current growing season stem and fine roots did not differ statistically significantly between treatments at final harvest (*P*_T_ ≥ 0.128), although starch and NSC contents were non-significantly 47–61% higher in Cool and EWLC than in Warm and ECLW (Figure 11e and f).

## Discussion

### Water relations and gas exchange

As expected, cool soil negatively affected tree water relations. After the soil temperature switch between 10 and 18 °C, the water potential of long-shoot leaves (the leaves emerging from the current year’s shoots) was lower in currently cool treatments (Cool, EWLC) than in currently warm treatments (Warm, ECLW). In a previous study, the water potential of long-shoot leaves of silver birch did not differ significantly between soil temperatures of 10 and 20 °C, but was higher at 5 than 20 °C (Aphalo et al. 2006). The authors suggested that the stomatal closure at a soil temperature of 5 °C prevented the lowering of leaf water potential, which led to the somewhat unexpected result. Here, the stomatal conductance was low in Cool, and increased in ECLW and decreased in EWLC after the switch, but apparently the closure was not complete enough to increase the water potential. Stem sap flow followed the same pattern as stomatal conductance and increased when the soil temperature was switched from 10 to 18 °C and decreased after the switch from 18 to 10 °C. If water is available in the soil, the stem sap flow rate is generally increased by the higher air and soil temperatures (Juice et al. 2016, Yan et al. 2019). The changes in the water relations were probably caused by the increased viscosity of water and the reduced permeability of root membranes to water in cool soil (Pallardy 2008).

Gas exchange depended on soil temperature, as was expected based on earlier studies (Aphalo et al. 2006). Net photosynthesis (*A*_max_), stomatal conductance (*g*_s_) and transpiration (*E*) were higher in currently warm treatments than in currently cool treatments before the soil temperature switch. After the soil temperature switch, *A*, *g*_s_ and *E* decreased in EWLC and increased in ECLW. In Warm, *A*, *E* and *g*_s_ were not as high after the switch, and they also showed a large decrease in ECLW between the two latest measurements in the late growing season, which may be related to the SD conditions. However, *A*_max_ decreased less than *E*, and therefore, WUE (=*A*_max_/*E*) was in Warm and ECLW double that in Cool and EWLC at the end of GS2, which may also be because of the SD conditions. The decrease in gas exchange in Warm was simultaneous with the decrease in the CCI of short-shoot leaves (the leaves emerging from the previous year’s shoots) after the switch time. Aphalo et al. (2006) reported that *A*_max_ and *g*_s_ were significantly higher at a soil temperature of 20 °C than at 5 and 10 °C, whereas the differences in *C*_i_ were not as prominent. The decrease of *A*_max_, *g*_s_ and *C*_i_ in warm soil after the switch time that were observed in our experiment were also seen in the study of Aphalo et al. (2006), but less prominently, possibly because the conditions were constant in that study. The decrease in *A*_max_ in EWLC after the switch appeared to be caused by stomatal closure, as both *g*_s_ followed the same pattern as *A*_max_. Near the end of the LD phase, foliar N content was highest in ECLW (Kilpeläinen et al., in press), which coincided with the highest photosynthetic rate after the soil temperature switch in ECLW. However, the fastest drop in photosynthesis from the LD to SD conditions occurred in ECLW. Stomata were closed more in the currently warm treatments than in the currently cool treatments in the SD conditions, but the photosynthesis continued, resulting in clearly lower *C*_i._ This suggests that the acclimation of the photosynthetic machinery was related to soil temperature.

The leaf water potential, *A*_max_ and *g*_s_ of aspen (*P. tremuloides*) were lower at 5 and 10 °C than at a soil temperature of 20 °C (Wan et al. 1999). Evidently, there are species- and case-specific differences in the response of photosynthesis to soil temperature. For example, in two conifer species, an increased root zone temperature enhanced CO_2_ uptake more in Scots pine than in Norway spruce, which may be related to higher N translocation to the shoot in pine and to the roots in spruce, and further to differences in the amount of Rubisco, which is an essential enzyme in photosynthesis (Vapaavuori et al. 1992). On the other hand, in Norway spruce, Scots pine and silver birch seedlings, increased soil temperature did not significantly affect the net photosynthesis and seedling biomass, because respiration increased concurrently with gross photosynthesis (Pumpanen et al. 2012).

Soil temperature rise increased the efficiency of photosystem II, as the *F*_v_/*F*_m_ and Δ*F*/*F*_m′_ increased most in ECLW after the soil temperature switch, whereas there was an opposite trend in EWLC. In addition, season, Δ*F*/*F*_m′_ was the highest in Warm in the late growing. Similar to our birch results, the *F*_v_/*F*_m_ of the current year needles in Norway spruce seedlings was lower in soil temperature 9 °C compared with 13, 18 and 21 °C (Repo et al. 2004). However, soil temperature did not have a significant effect on the *F*_v_/*F*_m_ of jack pine (*Pinus banksiana*) and white birch (*Betula papyrifera*) seedlings, but low soil temperature increased Δ*F*/*F*_m′_ in jack pine and decreased it in white birch (Zhang and Dang 2005).

**Table 3:** Leaf traits (mean ± SE) of silver birch seedlings in soil temperature treatments during the treatment growing season (GS2) (*n* = 4).

	GS2 day E35				GS2 day L77			
	Cool	Warm	ECLW	EWLC	Cool	Warm	ECLW	EWLC
Leaf area (cm^2^)	11.9a ± 0.7	25.0b ± 1.8	10.5a ± 0.6	17.0a ± 1.2	17.6 ± 2.8	20.1 ± 1.7	19.7 ± 1.7	14.6 ± 1.5
Lower epidermis cell area (μm^2^)	768 ± 52	890 ± 48	521 ± 15	758 ± 124	629 ± 48	526 ± 26	744 ± 79	587 ± 52
Glandular trichome index	0.291 ± 0.032	0.276 ± 0.028	0.270 ± 0.045	0.204 ± 0.032	0.517 ± 0.041	0.434 ± 0.038	0.368 ± 0.051	0.606 ± 0.055
Non-glandular trichome index	0.190 ± 0.048	0.305 ± 0.086	0.335 ± 0.117	0.378 ± 0.122	-	-	-	-
Leaf thickness (μm)	138 ± 6	156 ± 9	157 ± 10	158 ± 10	168 ± 2	174 ± 7	183 ± 9	164 ± 6
Upper epidermis thickness (μm)	17.2 ± 0.8	17.8 ± 0.8	18.8 ± 1.0	19.3 ± 1.2	21.7 ± 0.3	22.8 ± 1.0	22.2 ± 1.4	21.3 ± 1.0
Lower epidermis thickness (μm)	11.3 ± 0.5	13.0 ± 0.6	12.6 ± 0.5	11.7 ± 0.3	11.9a ± 0.5	14.9b ± 0.3	13.7b ± 0.5	14.0b ± 0.3
Palisade layer thickness (μm)	52.3 ± 3.0	51.8 ± 3.5	52.5 ± 3.2	46.6 ± 3.2	61.4 ± 1.9	63.8 ± 2.5	61.0 ± 3.4	66.2 ± 5.2
Spongy layer thickness (μm)	60.8 ± 3.4	74.9 ± 6.6	74.7 ± 6.7	84.2 ± 9.4	71.8ab ± 2.7	75.2ab ± 4.4	87.6b ± 6.4	63.9a ± 4.0
Palisade–spongy ratio	0.884 ± 0.053	0.748 ± 0.057	0.742 ± 0.037	0.596 ± 0.067	0.877 ± 0.046	0.880 ± 0.037	0.721 ± 0.068	1.078 ± 0.134

Short-shoot leaves were sampled on day E35, and long-shoot leaves on day L77. The epidermis cell area and the trichome indices were determined only at a lower leaf surface. Soil temperature was interchanged between cool (10 °C) and warm (18 °C) in treatments ECLW (early 10 °C, late 18 °C) and EWLC (early 18 °C, late 10 °C) between the sampling times. The different letters indicate significant differences between treatments (*P* ≤ 0.05).

**Table 4:** *P*-values of statistical tests of carbohydrate results (zero omitted before decimal separator).

	Short-shoot leaves			Long-shoot leaves		
Carbohydrate	Treatment	Time	Treatment × Time	Treatment	Time	Treatment × Time
Glucose	.995	.153	.314	.494	.151	**.007**
Fructose	.596	**<.001**	**.010**	**.020**	**<.001**	.113
Sucrose	**.014**	**<.001**	**.015**	.176	**<.001**	**.040**
Soluble sugars	.084	**<.001**	**.042**	.509	**<.001**	.114
Starch	**<.001**	**<.001**	**<.001**	**.002**	**<.001**	.088
NSC, total	**.003**	**<.001**	**<.001**	**.006**	**<.001**	.402

Values of *P* ≤ 0.05 are in bold. Post hoc test results are shown in respective figures.

We found that the CCI (depicts relative differences in chlorophyll content) of short-shoot leaves increased during the early growing season most in Warm and EWLC, but in the late growing season was ultimately lowest in Warm. The decrease of CCI in Warm accords with the decreasing gas exchange towards the late growing season. Leaf areas were largest in Warm, and a dilution effect in N, CCI and gas exchange per leaf area is possible. After the soil temperature switch, the temporal trend of CCI was the opposite in ECLW of that in EWLC. In long-shoot leaves, the CCI increased fastest in Warm and EWLC, and slowest in Cool and ECLW, where it was the highest at the end of the experiment. The CCI and foliar N findings (Kilpeläinen et al., in press) support each other as expected, because N is essential in chlorophyll construction. Aphalo et al. (2006) also concluded that a low soil temperature caused N starvation, leading to low chlorophyll content and reduced *A*_max_.

### Leaf anatomy

Warm soil increased leaf expansion and short-shoot leaves grew larger in Warm and EWLC than in Cool and ECLW. The first long-shoot leaves started to grow ca 1.5 weeks before the soil temperature switch, and therefore, their enlargement was not significantly affected by the switch. Instead, the long-shoot leaves that grew after the soil temperature switch were significantly larger in ECLW than in EWLC at the end of the experiment.

The hypothesis of a cool soil-induced secondary metabolism and increased defense got some support from the results concerning glandular trichome density, which was higher in cool than in warm soil. The largest difference was found in the long-shoot leaves between EWLC and ECLW. The hypothesis also gained some support from the glandular trichome index, which took the epidermis cell size into account. Glandular trichomes are generally costly to produce and can show a trade-off between vegetative and reproductive growths (Hare et al. 2003). Indeed, glandular trichome production and vegetative growth contradicted each other in our study, and the hypothesis of enhanced primary metabolism was materialized as largest tree biomass in Warm and enhanced shoot elongation in ECLW (Kilpeläinen et al., in press). However, the density of non-glandular trichomes was unaffected—they were not found at all in the long-shoot leaves in the latter half of growing season. Glandular trichomes store and excrete various secondary metabolites for defense against biotic and abiotic stresses (Oksanen 2018). For example, it has been shown that glandular, unlike non-glandular, trichomes are important for reducing leaf ozone uptake (Li et al. 2018). Both elevated air temperature and soil drought decreased the formation of glandular trichomes in the upper surface of silver birch leaves, but no interaction effects were observed (Thitz et al. 2017). The number of glandular trichomes in the lower leaf surface increased during the growing season, regardless of the treatments (Thitz et al. 2017). Trichome production in different environmental circumstances can therefore be complex.

The highest numbers of stomata per unit area (non-significant) and leaf (significant) were found in Warm. The increased stomatal density did not result in increased *A*_max_, which was lowest in Warm at the end of the LD phase (on day L80, leaves were sampled for anatomy on day L77). Later, during the SD phase (on day L94), WUE was highest in Warm and ECLW, although a lower stomatal density is usually associated with higher WUE (Bertolino et al. 2019). Opposite to our results, the stomatal density can decrease with increasing soil temperature, partly due to larger epidermis cell size and larger leaves, but stomatal ontogeny can also be directly affected by the soil temperature (Rogiers et al. 2011). The epidermis cell area had a pattern opposite to that of the stomata, but the epidermis cells were not significantly larger in warmer than cooler soil.

Based on an assumption of lower water supply from cool soil and thicker leaves preventing water loss (Larcher 2003), thicker leaves in cool than in warm soil could have been expected, but the data did not support this. Thick palisade mesophyll is connected with high photosynthetic activity, and thick spongy mesophyll with a large air space (Schollert et al. 2017). Thin palisade mesophyll and a high proportion of spongy mesophyll indicate poor photosynthetic potential in cool soil, but this was not supported by our results either.

The most remarkable change in glandular and non-glandular trichomes was in all treatments between the short-shoot and long-shoot leaves so that short-shoot leaves had more glandular trichomes but less non-glandular trichomes than long-shoot leaves. Net photosynthesis was about a third higher in long-shoot than short-short leaves, which accords with the observed higher stomatal density in long-shoot than short-shoot leaves. The leaf structures can thus be different in short-shoot and long-shoot leaves, and adjusted to different growing conditions during the growing season.

### NSCs

There was a tendency for carbohydrate content to increase in roots in cool soil, which accords with the slightly increased biomass allocation to roots too (Kilpeläinen et al., in press). The content of NSCs and starch as a large part of it were non-significantly up to 60% higher in Cool and EWLC than in Warm and ECLW at final harvest. Simultaneously, an opposite trend was visible in the stem of the current growing season, especially in ECLW. ECLW also stood out in the foliar NSC content, which declined after soil warming. An opposite pattern was recognized in EWLC, where the NSC content of short-shoot leaves started to increase with soil cooling. Thus, soil cooling may restrict silver birch growth more than according to the photosynthesis could have been expected. Similarly, the starch content of Norway spruce needles was greater at a lower than higher soil temperature, but root carbohydrates were not studied (Repo et al. 2004). At the end of our experiment, the NSC content of fine roots was also slightly and non-significantly higher in Cool and EWLC than in Warm and ECLW, which tends to confirm the observation of increased growth allocation to roots to enhance nutrient uptake in cool climates (Ostonen et al. 2011, Zadworny et al. 2016). The result is also in line with the surplus C theory that in constrained conditions, leaf growth is more suppressed than C assimilation would suggest, and surplus C is therefore partly translocated from leaves to roots, but also converted to secondary metabolites (Prescott et al. 2020). In the longer term, the pattern we observed in resource allocation may result in an increased root/shoot ratio in cool soil, which may further be enforced by increased fine root mortality in warm soil (Kilpeläinen et al., in press).

## Conclusions

To conclude, our results indicated significant soil temperature effects on the water relations, gas exchange, foliar anatomy and carbohydrate dynamics of silver birch. A soil temperature switch from low to high improved the water status, net photosynthesis, CCI, effective yield of photosystem II (Δ*F*/*F*_m′_), mobilization of NSCs and leaf area growth of silver birch seedlings. Soil cooling increased glandular trichomes. This investment in increasing chemical defense potential may be associated with decreased vegetative growth in cool soil. Warm treatment increased stomatal number per leaf, but it did not result in increased area-based gas exchange. Our findings added new information about the accumulation of NSCs in the foliage and roots of silver birch grown at a low soil temperature that may hinder vegetative growth more than net photosynthesis. Silver birch has two flushes of leaves during a growing season, and the leaf structure can be adjusted independently in the flushes to optimize fitness in prevailing growing conditions. Possible colder soils in future springs would firstly hamper the growth of both roots and shoots of silver birch (Kilpeläinen et al., in press), but simultaneously increase the chemical defense potential. However, silver birch would gain on the growth delay with the accumulated carbohydrates and increased availability of nutrients in the soil warming later in the growing season. The complex interactions of the structure and function of silver birch and the soil properties change along with soil temperature, which can further reflect to ecosystem functioning and carbon dynamics in boreal forests and back to climate change.

## Authors’ Contributions

J.K., T.D., T.L. and T.R. planned the study. J.K. measured leaf area and water potential and prepared and photographed the leaf replicas. T.D. measured gas exchange, M.K. the leaf anatomy variables from photos, and F.M. carbohydrates. J.K. performed statistical tests and wrote the first manuscript draft, and all the authors participated in writing the final manuscript.

## Data and Materials Availability

The data and materials are available to academic researchers on reasonable request.
